# Plastic Behaviour Buffers Climate Variability in the Wandering Albatross

**DOI:** 10.1002/ece3.70631

**Published:** 2024-11-28

**Authors:** Natasha Gillies, Jack Thorley, Henri Weimerskirch, Stéphanie Jenouvrier, Christophe Barbraud, Karine Delord, Samantha C. Patrick

**Affiliations:** ^1^ School of Environmental Sciences University of Liverpool Liverpool UK; ^2^ Department of Zoology University of Cambridge Cambridge UK; ^3^ Centre d'Études Biologiques de Chizé UMR 7273 du CNRS‐La Rochelle Université Villiers‐en‐Bois France; ^4^ Biology Department Woods Hole Oceanographic Institution Woods Hole Massachusetts USA

**Keywords:** climate, foraging, seabirds, southern annular mode, southern oscillation index, wandering albatross

## Abstract

Climate change has marked effects on global weather patterns and oceanic systems, impacting animal behaviour and fitness in potentially profound ways. Despite this, we lack detailed information about species' responses to climatic variation. Using an 11‐year tracking dataset of over 300 individual birds, we explore the consequences of variation in the southern annular mode (SAM) and southern oscillation index (SOI) for individual behaviour and fitness in wandering albatrosses *Diomedea exulans* breeding in the Southern Indian Ocean. Our results reveal distinct responses between males and females to climatic variation that align with the impacts of each climatic index on the distinct foraging ranges of each sex. In positive SAM phases, linked to poorer foraging conditions in female ranges and better conditions in male ranges, females exhibited behaviour consistent with reduced foraging success: that is, fewer prey capture attempts and more movement between feeding patches. Males, on the other hand, showed no behavioural change. During positive SOI phases, associated with good foraging conditions in both male and female foraging ranges, both sexes showed evidence of more successful foraging, with birds engaging in more search behaviour, and taking shorter trips with fewer prey capture attempts, together indicating increased food intake per unit time. We found limited evidence for a role of individual variation, as measured through differences in personality, suggesting that plastic responses to climate are sufficiently important so as to obscure inter‐individual variation. Supporting this was the finding that individual breeding success was unaffected by climatic variation, suggesting that plastic foraging behaviour allows albatrosses to mitigate climate impacts and maintain reproductive output.

## Graphical abstract

This study explores the impact of climate variation on the behaviour and fitness of wandering albatrosses using an 11‐year tracking dataset. The findings reveal that males and females respond differently to climatic changes, with females experiencing reduced foraging success during certain conditions, while males remain unaffected. Despite these behavioural shifts, the study found that individual breeding success was largely maintained, indicating that plastic foraging behaviour helps mitigate climate impacts.

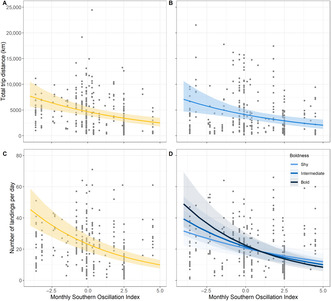

## 1 Introduction

Human impacts present a major threat to global biodiversity, with 20% of vertebrates now considered at risk of extinction, and climate change is recognised as playing a key role (Pereira et al. 2010; Bellard et al. 2012). Climate exerts profound influences on global weather patterns and oceanic systems, with significant downstream consequences for ecosystems and ecological dynamics. Understanding the ways in which animals respond to this is pertinent, particularly as significant increases in the intensity and frequency of extreme climate events are predicted for the coming decades (Easterling et al. 2000; Bailey and van de Pol 2016; van de Pol et al. 2017; Wang et al. 2022). Yet while mounting evidence is demonstrating effects of climate on phenology, demography, and behaviour across a wide range of species (Selwood, McGeoch, and Mac 2015; Buchholz et al. 2019; Inouye 2022; Lewin et al. 2024), neither the mechanisms underpinning these effects nor the relationships between them are fully resolved.

In the face of environmental change, animals have three options, often summarised as ‘adapt, move, or die’: that is, they can adapt genetically, adjust their distribution or behaviour, or face extinction (Wong and Candolin 2015). For many long‐lived vertebrate species, contemporary climate change may outpace genetic adaptation, leaving behavioural adjustment, such as through migratory range shifts (Lewin et al. 2024), changes in foraging effort (Speakman et al. 2021), or alterations to communication (Lengagne 2008), as the primary adaptive option. Behaviour therefore emerges as a crucial factor promoting species' ability to cope with long‐term environmental change. Behavioural plasticity, the ability of animals to adjust to environmental stimuli, varies considerably amongst individuals (Wilson 1998; Dall, Houston, and McNamara 2004; Nussey, Wilson, and Brommer 2007; Stamps 2016), with potential impacts on the long‐term persistence or trajectory of populations if subsets of populations cannot respond appropriately to changes in their environment. Understanding individual responses is therefore vital to predict species‐level responses to climate change. Personality traits—particularly ‘boldness’, which typically measures the responses of individuals to novel stimuli (Sih, Bell, and Johnson 2004; Patrick, Charmantier, and Weimerskirch 2013; Stamps and Biro 2016)—are increasingly recognised for their association with individual variation in behavioural plasticity (Dingemanse et al. 2010; Mathot and Dingemanse 2012; Stamps and Biro 2016; Gibelli and Dubois 2017). Shyer individuals are observed to be more responsive to environmental changes, while bolder individuals seem to be more fixed in their behaviours (Verbeek, Drent, and Wiepkema 1994; Groothuis and Carere 2005; Coppens, de Boer, and Koolhaas 2010; Adriaenssens and Johnsson 2011; Gibelli and Dubois 2017). Personality may therefore offer a valuable, yet understudied, metric to assess individual variation in the capacity of animals to adapt to a changing climate.

In the marine environment, climate has been found to play important roles in behaviour, survival, and fitness in a number of species including southern elephant seals *Mirounga leonina* (Volzke et al. 2021), emperor penguins *Aptenodytes forsteri* (Jenouvrier et al. 2012), and blue petrels *Halobaena caerulea* (Guinet et al. 1998). Amongst marine fauna, seabirds offer particularly valuable indicators of the state of marine environments due to the ease of measuring their behaviour and demography, responsiveness to prey availability, sensitivity to weather conditions, and adaptable behaviour (Frederiksen, Mavor, and Wanless 2007; Parsons et al. 2008; Durant et al. 2009; Mallory et al. 2010). Species that cover large foraging distances are of particular interest due to their exposure to large temporal and spatial environmental variation.

Seabirds demonstrate flexible adjustment to rapid environmental change, but there are likely to be limits to this, as shown at a population level by the limited advancement of breeding phenology across seabirds (Keogan et al. 2018), despite consistent directional selection favouring earlier breeding in many cases (Reed et al. 2009; Dobson et al. 2017; Descamps et al. 2019). Additionally, the presence of repeatable individual behaviour, possibly reflecting fixed behavioural strategies, may constrain responses to environmental change (Patrick and Weimerskirch 2014; Ceia and Ramos 2015; Krüger et al. 2019). Climate‐induced environmental changes can directly alter the energetic costs of behaviour, induce physiological stress, or lead to breeding failures or mass seabird wrecks during extreme weather events (Hass, Hyman, and Semmens 2012; Barbraud, Delord, and Weimerskirch 2015; Newell et al. 2015). Indirectly, changes in resource availability can have significant impacts. Seabirds are central‐place foragers while breeding, meaning they face spatial constraints in access to resources, which can lead to spatio‐temporal match‐mismatch between their movement and resource availability (Grémillet and Boulinier 2009). This can lead to reduced survival and breeding success when individuals struggle to maintain foraging effort or success (e.g., Cory's shearwater *Calonectris borealis*, Pereira et al. 2020; northern gannet *Morus bassanus*, Montevecchi et al. 2021; king penguins *Aptenodytes patagonicus* ; Le Bohec et al. 2008). Simultaneously, investigating the impacts of climate on behaviour and fitness is therefore essential to predict how seabirds will fare in the face of ongoing climate change (Jenouvrier 2013; Jenouvrier et al. 2018).

Wandering albatrosses *Diomedea exulans* exhibit some of the longest—in both distance and duration—foraging trips amongst seabirds (Weimerskirch et al. 2014), during which they show high levels of responsiveness to environmental variation (Weimerskirch et al. 2000; Richardson, Wakefield, and Phillips 2018) that varies amongst individuals (Gillies et al. 2023). Foraging behaviour differs markedly between the sexes, with males typically undertaking much longer trips to more southerly locations and being much more dependent on high wind speeds for efficient movement (Shaffer, Weimerskirch, and Costa 2001; Wakefield et al. 2009; Clay et al. 2020). Age also plays a role, with older males undertaking longer‐distance, more southerly foraging trips (Lecomte et al. 2010). Evidence that climate change may alter albatross behaviour is already emerging: increases in wind speeds in the southern ocean have been linked to improved breeding success, possibly due to reduced energetic costs when commuting during foraging flight (Weimerskirch et al. 2012). However, beyond wind, little is known about the potential impacts of environmental change on individual behaviour and breeding success in wandering albatrosses.

Disentangling the effects of environmental variation on behaviour, and ultimately, fitness, is complex. While single metrics such as sea surface temperature (SST) or air temperature have been linked to changes in behaviour and phenology (e.g., loggerhead turtles *Caretta caretta*, Mazaris et al. 2008; Western Australian Magpies *Cracticus tibicen dorsalis*, Edwards, Mitchell, and Ridley 2015), such measures offer only partial perspectives and capture relatively small‐scale variation—in time, space, or both (Stenseth et al. 2002; Le Bohec et al. 2008). Climate indices offer consolidated measures that capture much broader variation in the environment, integrating information over large spatial and temporal scales. While this may come at a cost to fully understanding causative pathways of effects, such broad understanding can inform predictions about future responses to climate change, help identify potential adaptive responses and vulnerabilities, and provide a foundation for more detailed mechanistic studies, improving our ability to predict the impacts of expected future change.

Across the southern ocean, the leading modes of climate variation are the Southern Annular Mode (SAM) and Southern Oscillation Index (SOI; Rogers and van Loon 1982; Thompson and Wallace 2000; Fogt and Marshall 2020). SAM describes variability in the strength and position of a belt of westerly winds encircling the Antarctic, which contracts towards and expands away from the south pole during positive and negative phases, respectively (Lovenduski and Gruber 2005; Fogt and Marshall 2020), with pronounced consequences for the wider environment dependent on latitude. Positive SAM phases enhance wind strength and upwelling south of the Antarctic Polar Front (approximately 50°–60° S), where male wandering albatrosses primarily forage, while north of this, where females forage, the effects are opposite (Lovenduski and Gruber 2005). The Southern oscillation index measures sea level pressure differences between Tahiti and Darwin, Australia (Wang et al. 2016, 2022). Positive SOI, which at an extreme may indicate La Niña events, is linked to positive zonal wind stress—indicating strong westerly winds, decreases in SST, and increased upwelling across the southern ocean (Newell, Selkirk, and Ebisuzaki 1982; McPhaden, Santoso, and Cai 2020; Wang et al. 2022). Very negative values indicate El Niño events, a warming phase associated with decreased upwelling and weakened winds. Patterns of SAM and SOI are expected to change in the coming decades: climate models predict more frequent and intense El Niño/La Niña events and shifts in the intensity and position of the westerly winds associated with the SAM (Easterling et al. 2000; Fogt and Marshall 2020; McPhaden, Santoso, and Cai 2020; Wang et al. 2022). Changes to both indices will have significant effects on oceanographic, atmospheric, and meteorological conditions.

We aimed to investigate how individual wandering albatrosses respond to SAM and SOI conditions and whether this affects their reproductive output. Changes in the SAM and SOI in the southern ocean have profound impacts on ocean currents and weather patterns at large spatial scales, which we anticipated would have behavioural consequences. Using a 11‐year tracking dataset from the Crozet Islands in the Southern Indian Ocean, we investigated how changes in the SOI and SAM related to breeding success and foraging behaviour. By incorporating personality, a fixed trait known to constrain plasticity, we explored consistent individual variation in responses to these indices. By comparing individual responsiveness and breeding success changes, we aimed to indirectly assess how climate affects foraging behaviour and subsequent reproduction, giving essential insights into the capacity of albatrosses to buffer environmental variation and future climate change.

## 2 Methods

### 2.1 GPS Tracking

We tracked the movements of 346 wandering albatrosses (175 males, 171 females) during the incubation period (January to April) of 2010 to 2021. Albatrosses were sampled from Possession Island (Crozet Islands archipelago, Southern Indian Ocean, 46°24′ S, 51°46′ E). Since 1965, each year all adults and chicks in the study population have been captured by hand and equipped with a metal leg‐ring and a plastic leg‐ring bearing a unique identification number (Weimerskirch 2018). Adults are sexed based on size and plumage dimorphism within breeding pairs (Weimerskirch, Lallemand, and Martin 2005).

Each albatross was fitted with a GPS logger (IgotU 120/600, Mobile Action Technology, weighing up to 32 g, max 0.5% body mass; X‐GPS and Centurion, Sextant Technology, NZ weighing 60–75 g, max 1.21% body mass; see details in Weimerskirch et al. 2018; Weimerskirch et al. 2020), which was deployed dorsally using thin strips of marine Tesa tape (Weimerskirch et al. 2014) and retrieved after the bird had completed at least one complete foraging trip. There is presently no evidence for an effect of such loggers on survival probability or breeding success in wandering albatrosses (Barbraud and Weimerskirch 2012). GPS loggers recorded fixes at frequencies ranging from 1 to 15 min, and the resulting data were resampled to give fixes at 15‐min intervals.

### 2.2 Measuring Boldness

Every year, the ‘boldness’ of all birds in the Possession Island colony is measured by observing how individuals react to the approach of a human observer (see (Patrick, Charmantier, and Weimerskirch 2013) for full details). The observer noted each bird's behaviours as they approached from a 5‐m distance, stopping short ofreaching the bird itself. Behaviours were recorded using a 5‐point ordinal scale ranging from 0 to 4, where 0 = no response; 1 = bird lifts head; 2 = bird rises onto tarsi; 3 = bird vocalises; 4 = bird stands up. Using this scale, higher scores indicate bolder birds. Using these measurements, we estimated boldness by extracting individual‐level best linear unbiased predictors from an ordinal generalised linear mixed model (GLMM) that was fitted to boldness scores using the R package MCMCglmm (Hadfield 2010). The model has been used in previous studies of this population (Patrick, Charmantier, and Weimerskirch 2013; Gillies et al. 2023), and full details on the methodology can be found therein. The model included the fixed effects of observation number and observer ID (typically one observer per year), a random intercept for individual ID, and a random effect for the additive genetic variance, represented as the matrix of pairwise relatedness amongst all individuals.

### 2.3 Climate Variables

Data on SAM indices were accessed from the National Center for Atmospheric Research Climate Data portal (https://legacy.bas.ac.uk/met/gjma/sam.html) on 2022‐01‐20 (Marshall 2003). Monthly SAM indices were calculated as numerical values representing the differences in monthly zonal sea level pressure at 40° S and 60° S. The data are observation‐based, collected from six monitoring stations at each latitude. Monthly SOI indices were downloaded from the National Weather Service—Climate Prediction Center on 2022‐01‐20 (National Weather Service—Climate Prediction Center 2022). SOI is calculated as the standardised difference between anomalies in sea level pressure between Tahiti and Darwin, Australia, normalised by the monthly standard deviation. Further details are available in the Appendix 1. SAM and SOI were not significantly correlated over the study period (Appendix 1).

### 2.4 Statistical Analysis

All data processing and statistical analyses were carried out in R version 4.3.0 (R Core Team 2023). Effects are presented as mean and [95% confidence interval] or mean ± standard deviation, unless otherwise specified. We interrogated fitted models by conducting diagnostic checks, including visual examination of residuals for normality, patterns, or trends, and assessing overdispersion using dispersion ratio checks.

#### 2.4.1 Effect of Climate on Behaviour

We aimed to determine how climate indices influenced albatross foraging behaviour and reproductive success. First, we fitted a three‐state hidden Markov model (HMM) to the GPS tracking dataset using the R package momentuHMM (McClintock and Michelot 2018) in order to categorise 15‐min fixes GPS into the three discrete behavioural states: rest, travel, and search. Models were fitted to males and females separately due to their known differences in movement behaviour (Shaffer, Weimerskirch, and Costa 2001; Clay et al. 2020; Gillies et al. 2023). The step length and turning angle were used as input variables, modelled with a Von Mises and a Gamma distribution respectively, and previous studies that made use of this dataset were used to guide parameterisation (Clay et al. 2020; Gillies et al. 2023). Using these inputs, the model identified rest as fixes with low speeds and low to moderately concentrated turning angles, search as fixes with moderate speeds and moderate to wide turning angles, and travel as fixes with high speeds and concentrated turning angles.

From these three broad behavioural categories, we calculated metrics of foraging behaviour that would most likely to be shaped by climate: the number of landings per day of the foraging trip, the ratio of time spent in search relative to travel, the total distance covered over the trip in kilometres, and the median distance travelled between search bouts (justification in Appendix 1). We fitted GLMMs to each metric, with the fixed effects of monthly climate index (SAM or SOI), boldness, and their interaction using the glmmTMB package in R (Brooks et al. 2017). We controlled for age, which is also known to affect foraging behaviour in wandering albatrosses (Lecomte et al. 2010; Froy et al. 2015), by including it as a fixed effect. Models were fitted separately for each sex and climate index (SAM or SOI) to reduce model complexity and therefore aid convergence and interpretation. All models included individual ID and year as random effects to account for repeated measures of the same individuals over time and annual variation, respectively. Total distance and median distance between search bouts was modelled with a Gamma distribution with a log link to reflect the positive right skewed nature of the response. The ratio of search relative to travel was modelled with a beta distribution due to its proportional nature. As a discrete count variable, number of landings per day was modelled with a Poisson distribution.

#### 2.4.2 Effect of Climate on Reproductive Success

We next examined the relationship between climatic variation and breeding to determine whether effects of climate on reproduction might be indirectly mediated by effects on foraging behaviour. Existing literature suggests that either SAM and SOI could have impacts prior to, during the breeding season, or both. We therefore averaged the monthly values of each index for September and November (pre‐breeding) and January to April (breeding). December was excluded as a mixture of pre‐breeding and breeding activity takes place during this month.

We considered available breeding success data for the entire Possession Island population, gathered between 1980 and 2020 (3052 individuals), to maximise available variation in the SAM and SOI. We fitted binomial GLMMs to the binomial variable current ‘breeding success’ (success vs. failure), which considered only those birds that attempted to breed. To reduce model complexity, each climate index was fitted in a separate model. Models included the fixed effects of mean breeding and pre‐breeding climate index (SAM or SOI respectively), age, and boldness. Interactions between age and each climate index (breeding and pre‐breeding) were included to account for the idea that older or younger individuals may be differentially equipped to cope with environmental variation; a further three‐way interaction was included between age, climate index, and boldness to account for the additional effect that boldness may have on this relationship. Non‐significant interaction terms were removed from the model to ensure accurate appraisal of fixed effects (Engqvist 2005). Age was incorporated as a quadratic predictor due to its known curvilinear association with breeding success (Weimerskirch 1992). All variables were centred and scaled to aid model convergence and interpretation of effects. Models were fitted for each sex separately due to known sex‐related differences in responses to the environment (Weimerskirch et al. 2018; Clay et al. 2020) and to avoid the involvement of complex four‐way interactions. Individual ID and year were included as random effects to control for repeated individual measures and interannual variation, respectively.

### 2.5 Ethics

All handling and experimental procedures were conducted in accordance with guidance and rules issued by the Réserve Nationale des Terres Australes. All field protocols and manipulations were granted approval by the Comité National de la Protection de la Nature and the ‘Préfet of Terres Australes et Antarctiques Françaises’ to Program IPEV No109.

## 3 Results

**FIGURE 1:**
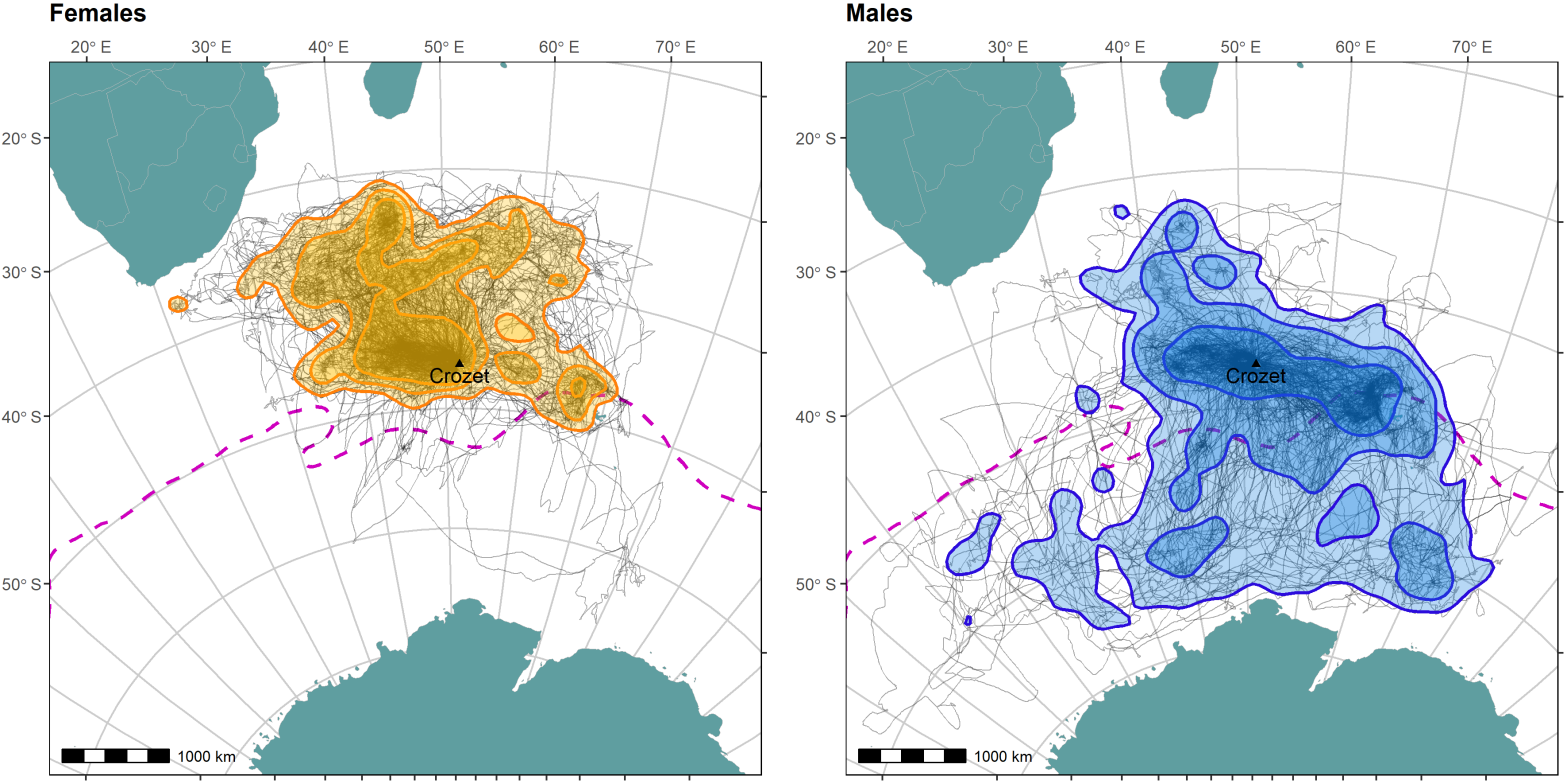
Foraging tracks of wandering albatrosses *Diomedea exulans* tracked during the study. Possession Island indicated with a black triangle. Grey tracks show individual bird movements; shaded polygons indicate 90% (lightest), 75% (mid), and 50% (darkest) utilisation distributions for all females (yellow polygons) and males (blue polygons). Pink dotted line shows approximate location of Antarctic Polar Front (Orsi and Harris 2019). Map and GPS tracks displayed in a Lambert azimuthal equal‐area projection, centred on Possession Island.

We obtained 690 foraging trips, giving an average of 2.02 ± 1.41 trips per bird. Trips lasted a mean 8.1 ± 5.7 days. As previously reported (Weimerskirch et al. 2012), females had more northerly foraging distributions compared to males (Figure 1).

**FIGURE 2:**
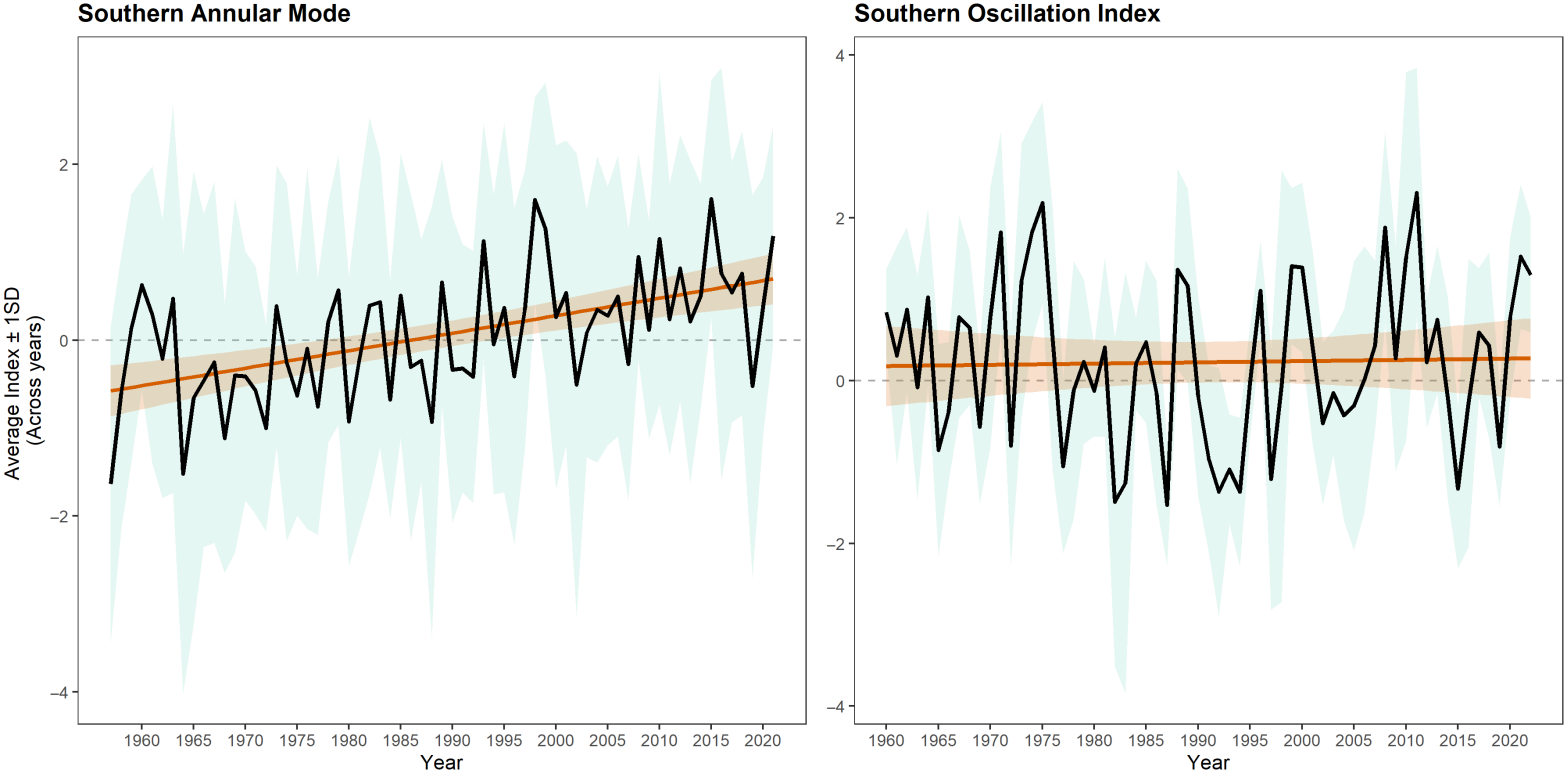
Temporal trends in the Southern Annular Mode and Southern Oscillation Index. Black line shows changes in the annual average for each index; green‐blue shading indicates standard error. Orange line and orange shading indicate regression and associated 95% confidence interval of change in index over time.

Over the past 60 years, SAM has shown a gradual tendency to become more positive over time (*β* = 0.02 ± 0.004, *t* = 5.11, *p* < 0.001), while SOI has been relatively stable (*β* = 0.002 ± 0.007, *t* = 0.22, *p* = 0.83; Figure 2). The range of SAM and SOI values observed during the study period (SAM: −2.1, 4.9; SOI: −3.6, 4.5) was broad but did not encompass the most extreme negative indices observed over the range of the past 60 years (SAM: −7.7, 4.9; SOI: −6, 4.8).

### 3.1 Effect of Climate on Behaviour

While changes in the SOI were found to have significant effects on foraging behaviour for both males and females, only females were affected by changes in the SAM.

**FIGURE 3:**
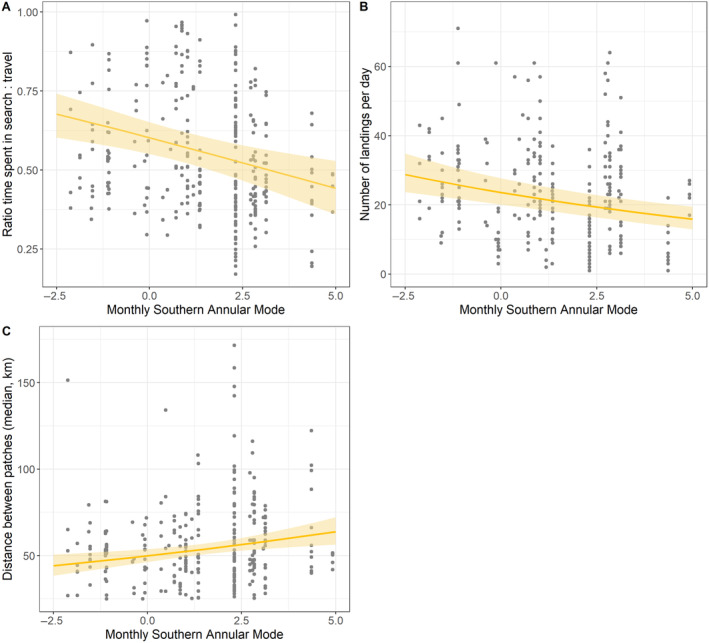
Effects of monthly southern annular mode (SAM) on (A), time spent in search relative to travel; (B) number of landings per day of the foraging trip; and (C) total distance travelled during foraging trips (km) for females. Lines and shaded areas indicate the regression and 95% confidence intervals for the effect of SAM. Each data point represents a single foraging trip.

**FIGURE 4:**
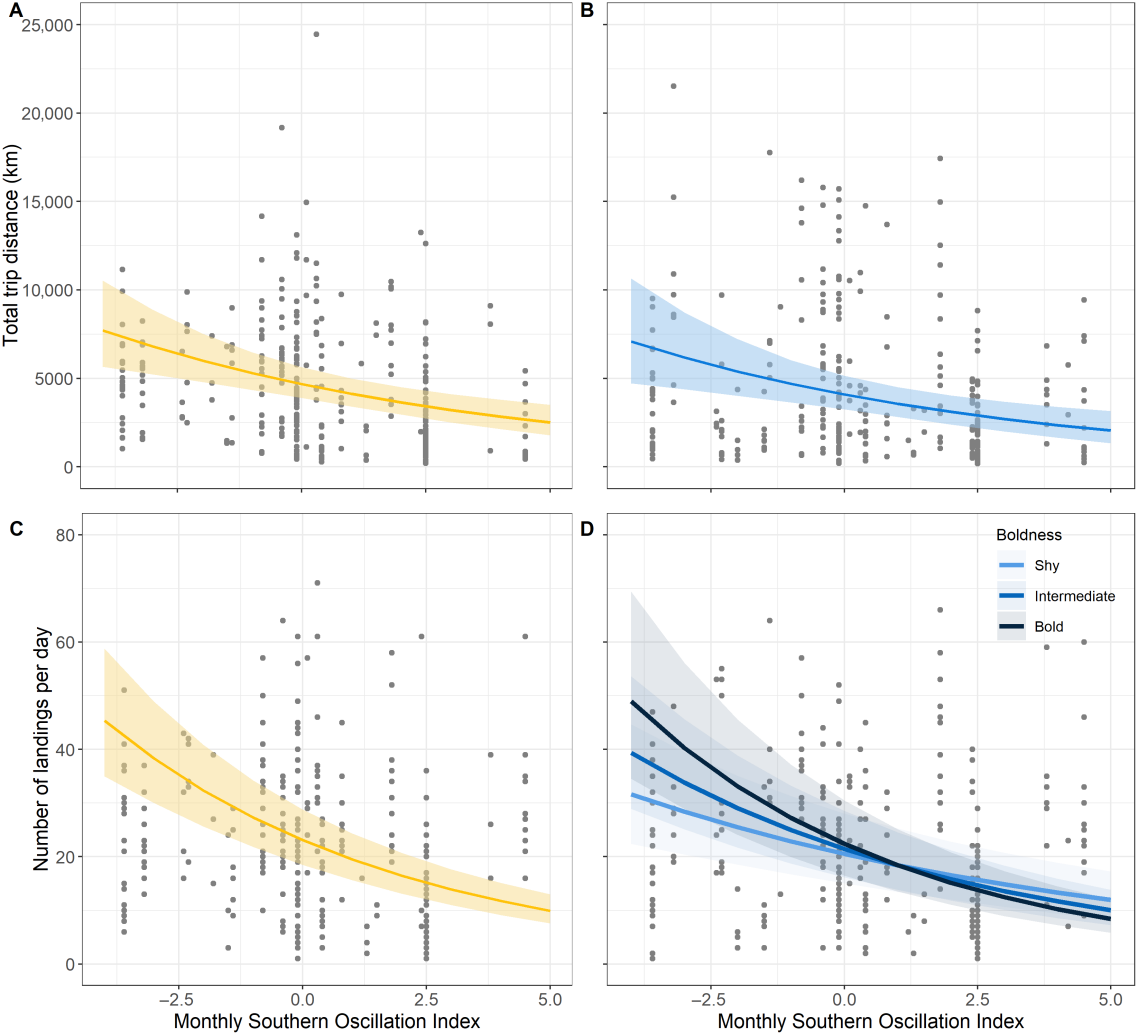
Effects of monthly southern oscillation index (SOI) on (A, B) total distance travelled during foraging trips (km) and (C, D), number of landings per day of the foraging trip for females in yellow and males in blue. Lines and shaded areas indicate the regression and 95% confidence intervals for the effect of SOI. Male responses in panel D are categorised by boldness, with lighter lines for shyer birds (10th percentile), intermediate lines for moderate birds (50th percentile), and darker lines for bolder birds (90th percentile). Each data point represents a single foraging trip.

Females decreased the number of landings they made per day with increases in SAM and SOI (Figures 3 and 4); males decreased their number of landings in response to SOI only (Figure 4, Table 1). For a one‐point increase in SAM, females made 8.22% fewer landings; for a one‐point increase in SOI, they made 15.52% fewer. Male responses to SOI depended on boldness. Shyer males had a reduced response to SOI, reducing landings by 10.24% for a one‐point increase in the SOI, while bolder birds reduced landings by 17.76%. Overall, bolder males made more landings per day, with a mean of 23.7 landings per day versus 20.4 for shyer males.

**TABLE 1:** Model estimates from the set of generalised linear mixed effects models examining the effect of the southern annular mode (SAM) and southern oscillation index (SOI) on foraging behaviour.

Response	Predictor	Estimate (odds ratio^a^) and 95% confidence interval		*z* value		*p*	
		*F*	*M*	*F*	*M*	*F*	*M*
Number of landings	***SAM***	***0.92 [0.89, 0.95]***	1.01 [0.98, 1.04]	***−4.80***	0.54	***< 0.001***	0.59
	Boldness	1.07 [0.99, 1.16]	1.01 [0.94, 1.09]	1.63	0.27	0.10	0.78
	Age	1.00 [0.99, 1.01]	1.01 [1.00, 1.02]	0.00	1.59	0.99	0.11
	**SOI**	**0.84 [0.82, 0.87]**	**0.85 [0.83, 0.88]**	**−10.41**	**−10.25**	**< 0.001**	**< 0.001**
	Boldness	1.07 [1.00, 1.15]	1.03 [0.96, 1.10]	1.84	0.71	0.066	0.48
	Age	1.00 [0.99, 1.01]	1.01 [1.00, 1.02]	−0.06	1.72	0.95	0.085
	***SOI × boldness***		***0.97 [0.96, 0.99]***		***−3.42***		***0.001***
Ratio search: travel	**SAM**	**0.8 [0.82, 0.94]**	**0.95 [0.90, 1.00]**	**−3.55**	**−1.94**	**< 0.001**	**0.003**
	Boldness	1.00 [0.92, 1.09]	0.95 [0.87, 1.03]	0.03	−1.23	0.98	0.053
	Age	1.00 [0.99, 1.01]	1.00 [0.99, 1.01]	−0.42	0.23	0.67	0.82
	SOI	1.03 [0.93, 1.13]	1.02 [0.97, 1.08]	0.57	0.78	0.57	0.43
	Boldness	0.99 [0.91, 1.08]	0.94 [0.87, 1.03]	−0.16	−1.37	0.87	0.17
	Age	1.00 [0.99, 1.01]	1.00 [0.99, 1.01]	−0.11	0.44	0.92	0.66
Total path distance (km)	SAM	1.04 [0.96, 1.12]	1.05 [0.97, 1.14]	1.00	1.26	0.32	0.21
	Boldness	1.04 [0.95, 1.15]	1.04 [0.94, 1.15]	0.92	0.74	0.36	0.46
	Age	1.00 [0.99, 1.01]	1.01 [1.00, 1.03]	−0.39	1.56	0.70	0.12
	**SOI**	**0.88 [0.83, 0.94]**	**0.87 [0.81, 0.94]**	**−4.16**	**−3.49**	**< 0.001**	**< 0.001**
	Boldness	1.05 [0.96, 1.15]	1.04 [0.94, 1.15]	1.14	0.82	0.25	0.41
	Age	1.00 [0.99, 1.01]	1.01 [1.00, 1.02]	−0.13	1.62	0.90	0.10
Median distance between search patches (km)	***SAM***	***1.05 [1.02, 1.08]***	1.03 [1.00, 1.07]	***3.06***	1.84	***0.002***	0.066
	Boldness	0.98 [0.94, 1.03]	1.02 [0.97, 1.07]	−0.69	0.91	0.49	0.36
	Age	1.00 [0.99, 1.00]	1.00 [1.00, 1.01]	−0.37	1.49	0.71	0.14
	SOI	1.00 [0.97, 1.02]	1.00 [0.97, 1.02]	−0.33	−0.20	0.74	0.85
	Boldness	0.99 [0.95, 1.04]	1.02 [0.98, 1.07]	−0.34	0.98	0.74	0.33
	Age	1.00 [0.99, 1.00]	1.00 [1.00, 1.01]	−0.86	1.34	0.39	0.18

*Note:* Total path distance was fitted with a Gamma distribution (log link); ratio search: Travel with a beta distribution, and landings per day with a Poisson distribution (log link): Estimates are provided on the relevant link scale. An odds ratio of 1 suggests no effect, an odds ratio > 1 suggests a positive association, and an odds ratio < 1 suggests a negative association. Significant *p* values and associated beta estimates and test statistics are highlighted in bold if they were found to be significant in both sexes; they are highlighted in bold and italics if they were found to be significant in only one sex. Non‐significant interactions were dropped.

^a^ Estimate for number of landings is an incidence rate ratio due to Poisson distribution.

The amount of time females spent in search behaviour relative to travel was affected by SAM conditions but not the SOI; males were unaffected by either index (Figure 3, Table 1). In positive SAM conditions, females reduced their search time relative to travel; a one‐point increase in the SAM was associated with a 5.49% decrease in time spent in search relative to travel.

We found that the total distance covered by birds on foraging trips decreased as SOI increased (Figure 3, Table 1). A one‐point change in the SOI was associated with a 13.31% decrease in travel distance for females and a 14.76% change for males. There was no effect of SAM on the total travel distance for either sex. However, for females, a one‐point change in the SAM was associated with a 5.03% increase in the distance travelled between search bouts.

### 3.2 Effect of Climate on Reproductive Success

**FIGURE 5:**
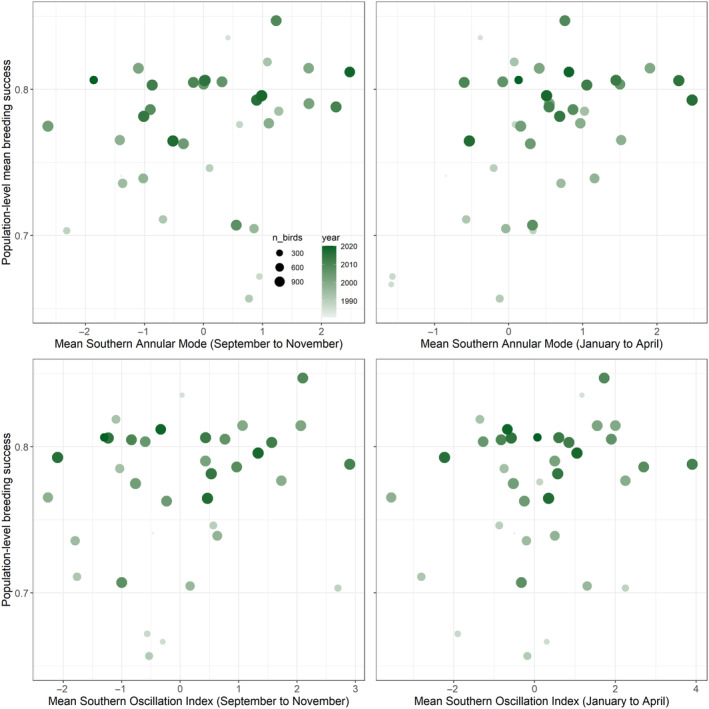
Breeding success as a function of changes in the southern annular mode and southern oscillation index during breeding and pre‐breeding periods. Points indicate mean breeding success for each year (labelled); size indicates sample for each year. Older year boxes coloured in lighter shades of green, more recent years in darker shades.

Mean breeding success for the entire Possession Island study colony between 1980 and 2020 was high at 77.96% ± 3.93% (mean weighted by annual sample size) and has been increasing annually (Table 2). We found no evidence for effects of SAM or SOI on individual breeding success for either sex and no effect of boldness (Figure 5, Table 2). The relationship between age and breeding success was quadratic, with breeding success gradually increasing until around 25–30 years, at which point it declined.

**TABLE 2:** Model estimates from binomial generalised linear mixed effects models examining the effect of the southern annular mode (SAM) and southern oscillation index (SOI), averaged (mean) for the breeding and non‐breeding periods, on individual reproductive success.

Predictor	Odds ratio and 95% confidence interval				*z* value				*p*			
	SAM		SOI		SAM		SOI		SAM		SOI	
	*F*	*M*	*F*	*M*	*F*	*M*	*F*	*M*	*F*	*M*	*F*	*M*
Age	**1.23 (1.13, 1.33)**	**1.19 (1.10, 1.29)**	**1.23 (1.13, 1.33)**	**1.20 (1.11, 1.30)**	**4.90**	**4.33**	**4.94**	**4.50**	**< 0.001**	**< 0.001**	**< 0.001**	**< 0.001**
Age 2	**0.82 (0.78, 0.87)**	**0.85 (0.81, 0.90)**	**0.82 (0.78, 0.87)**	**0.85 (0.81, 0.90)**	**−7.18**	**−5.98**	**−7.20**	**−6.00**	**< 0.001**	**< 0.001**	**< 0.001**	**< 0.001**
Mean SAM pre‐breeding	0.98 (0.91, 1.05)	1.03 (0.94, 1.13)	1.06 (0.91, 1.23)	1.10 (0.92, 1.33)	−0.59	0.56	0.74	1.05	0.55	0.58	0.46	0.30
Mean SAM breeding	1.07 (0.99, 1.15)	1.06 (0.96, 1.18)	1.00 (0.86, 1.17)	1.00 (0.83, 1.20)	1.79	1.20	0.05	−0.03	0.073	0.23	0.96	0.98
Boldness	0.94 (0.87, 1.03)	0.97 (0.90, 1.05)	0.94 (0.86, 1.02)	0.97 (0.90, 1.05)	−1.36	−0.72	−1.40	−0.70	0.18	0.47	0.16	0.48

## 4 Discussion

Over an 11‐year period, we observed that climatic variation significantly impacted the behaviour of wandering albatrosses, yet did not impact their breeding success, suggesting that albatrosses are able to buffer the effects of climate on their foraging ecology to preserve their reproductive success. This work adds to a growing body of evidence suggesting that behavioural plasticity may be an essential mechanism by which animals can adjust to the changing environment and ultimately buffer the negative impacts of widescale climatic change (Bradshaw and Holzapfel 2006; Charmantier et al. 2008; Grémillet and Boulinier 2009; Moritz and Agudo 2013).

Variation in SAM differentially impacted the foraging ranges of female and male albatrosses. Positive SAM phases are associated with less favourable conditions for female foraging, marked by weaker winds and increased sea surface temperature (see figure 1 in Lovenduski and Gruber 2005) and in such conditions females were found to land less frequently and to increase their travel time relative to searching. Landing is associated with prey capture attempts (Weimerskirch, Wilson, and Lys 1997; Weimerskirch and Guionnet 2002), suggesting that females were making fewer attempts to capture prey items. Combined with observations that both relative travelling behaviour and the distance travelled between bouts of searching behaviour increased, this suggests that females were encountering fewer feeding patches during positive SAM phases. Conversely, the positive SAM phase in male foraging ranges correlates with improved foraging conditions—specifically, increased wind speeds, cooler SST, and possibly increased chlorophyll levels. Despite this, male behaviour did not show adjustment in response to SAM. This may be due to the spatial variability of SAM effects, which tend to be beneficial in the south but disadvantageous in the north. As Figure 1 illustrates, males forage both north and south of the Antarctic Polar Front and so could experience improved or diminished foraging conditions depending on their chosen foraging location, potentially masking an overall relationship with foraging success. Alternatively, it is possible that this dichotomy emerges from between‐sex differences in plasticity, a pattern that has been observed across a very small but a diverse, range of other species (Bonier et al. 2007; Meuthen et al. 2018; Brand et al. 2023). It is difficult, however, to disentangle this from the fact that females are also probably exposed to greater variation in foraging habitat quality as they typically forage north of the Antarctic Polar Front, where oceanographic conditions are more variable. Conditions in male foraging grounds are generally good for foraging, being characterised by stronger winds and cooler temperatures, and so further positive change may not have had detectable effects (Wakefield et al. 2009; Clay et al. 2020). The increasing trend for positive SAM in recent years may further obscure effects on male behaviour (Figure 3; Abram et al. 2014; Fogt and Marshall 2020).

Variation in SOI affects female and male foraging ranges similarly, with positive phases correlating with cooler SST and stronger winds, the former being thought to indicate better foraging conditions while the latter makes at‐sea travel more efficient (Richardson, Wakefield, and Phillips 2018; Evans, Lea, and Hindell 2021). During positive SOI phases, both sexes showed behaviours that in combination may indicate successful foraging, that is, fewer landings and shorter foraging distances (thought to be indicative of successful trips; Salamolard and Weimerskirch 1993; Shaffer, Costa, and Weimerskirch 2003; Weimerskirch et al. 2007). Females additionally spent more time in search behaviour. These responses may suggest that birds were experiencing more successful foraging trips, making fewer prey capture attempts that were either more successful or resulted in the capture of more or larger prey. While it is unclear why males responded to SOI variation, but not SAM, the greater variability in SOI during the study period may have made its effects more detectable as compared to SAM.

By incorporating the metric ‘boldness’ in our analyses, we aimed to quantify the role of individual variation in responses to climate. Overall, boldness had a limited impact on individual behavioural responses to longer‐term environmental change: personality was only found to be important in male responses to the SOI, where shy males were found to adjust their number of landings to a lesser extent than bolder males, contrasting with theoretical work predicting that shyer individuals should be more attuned to their environment (Mathot and Dingemanse 2012; Snell‐Rood 2013; Stamps 2016; Gibelli and Dubois 2017), though this effect was modest. Female responses did not depend on personality, a surprising result given that previous work indicates that boldness influences short‐term behavioural responses to specific environmental factors, such as instantaneous wind conditions, in female wandering albatrosses (Gillies et al. 2023). This is unlikely to reflect a lack of perceived ‘risk’ as birds experienced a full spectrum of environmental conditions, which should include both ‘risky’ and ‘non‐risky’ scenarios. Instead, this discrepancy indicates that while personality traits may affect immediate, short‐term behavioural adjustments, they might not significantly impact long‐term responses to broader climatic factors. This is consistent with previous findings that boldness has a minimal impact on female demographic rates (Van de Walle et al. 2024) and overall breeding success (Gillies et al. 2023). These findings support the idea that behavioural plasticity is a crucial mechanism by which species buffer potential impacts of environmental variation on their fitness (Komers 1997; Chevin, Lande, and Mace 2010; Beever et al. 2017).

As albatrosses are long‐lived and thus prioritise self‐maintenance over reproductive investment, poor foraging conditions during the breeding season could negatively impact their offspring's survival and development through reduced care or provisioning quality (Stearns 1992). Despite this, we found no effect of SAM or SOI on individual breeding success. It is possible that negative impacts on female foraging were offset by the success of their male partners, given the contrasting impacts on their respective foraging ranges. Alternatively, or additionally, albatrosses may have mechanisms to mitigate environmental impacts before they impact reproductive output. As capital breeders, the costs of reproduction in wandering albatrosses are likely to be paid from resources accumulated prior to breeding, with resources gained during the breeding season being allocated to maintaining parental condition rather than being passed to offspring (Jonsson 1997). Indeed, previous work has shown that albatrosses experiencing improved foraging conditions during provisioning do not invest this extra energy into their chick (Berrow and Croxall 2001). However, our measure of breeding success considers only chick survival until fledging and so may not capture long‐term fitness impacts. Previous research indicates potential cohort effects due to environmental variation (Fay et al. 2015, 2016), highlighting the need for additional measures such as chick body condition and recruitment to fully understand the fitness impacts of climatic variation on albatrosses. Furthermore, conditions during the pre‐breeding season can have significant consequences for the probability of attempting to breed, even if it does not have effects on breeding success directly (Van de Walle et al. 2024). Future studies should explore potential long‐term effects on reproduction, including impacts on offspring quality, breeding frequency and probability, or reproductive lifespan, given known carry‐over effects from current to future reproduction in seabirds (Catry et al. 2013; Fayet et al. 2016; Moe et al. 2017; Harris et al. 2020).

Ultimately, the finding that breeding success was unaffected by climatic variation may suggest that all individuals are selected to behaviourally buffer this variation, at least within a season. This may additionally help explain the finding that personality did not have strong effects on behaviour, if responses to climate are so conserved that they mask any potential effects of boldness. However, our study captures only an 11‐year snapshot of climatic variation, during which time the most extreme negative values of SAM and SOI were not observed. Wandering albatrosses are capable of considerable behavioural feats, travelling several thousand kilometres in a single foraging trip and so are adept at behaviourally buffering variation, something that has also been observed in their responses to long‐term changes in wind conditions (Weimerskirch et al. 2012). However, there are almost certain limits to the environmental variation that birds can withstand. Indeed, previous work has found that wandering albatrosses do have a maximum tolerable wind speed that they act to avoid (Nourani et al. 2023), suggesting that more extreme variation could in theory lead to individual differences in responses that we were unable to capture. Furthermore, as the breeding season progresses, the ability to buffer environmental variation may become particularly critical as the chick hatches and the energetic demands of provisioning therefore increase. Such demands may restrict the capacity of albatrosses' behavioural coping mechanisms, which could significantly affect foraging efficiency and, consequently, reproductive success.

Our study sheds light into the relationship between climatic variation, foraging behaviour, and reproductive success in wandering albatrosses. While we observed significant impacts of changes in SAM and SOI on foraging behaviour, these did not translate into effects on reproductive success. This suggests that the behavioural plasticity exhibited by seabirds, including albatrosses, may provide some protection against the broader effects of climate warming. However, it remains unclear whether environmental effects may still impact overall fitness or whether small effects that are undetectable at this scale could accumulate over albatrosses' entire lifecycles. Moreover, this plasticity is unlikely to be limitless (Somveille et al. 2020), particularly as climate variability intensifies, exposing animals to extremes of environmental variation to which they cannot adapt. Seabirds have already experienced mass die‐offs, wrecks, and heat stress associated with extremes of wind and temperature (Lempidakis et al. 2022; Nourani et al. 2023). Similar trends have been observed for terrestrial species when extreme conditions exceed the capacity of species to behaviourally adjust or lead to the expression of catastrophically maladaptive behaviour (Santini et al. 2016; van Baaren and Candolin 2018; Sharpe, Bayter, and Gardner 2021). Such effects may be compounded by anthropogenic impacts such as fishing, introduced predators, land‐use change, or pollutants (Thuiller et al. 2006; Goutte et al. 2014; Oro 2014; Barbraud et al. 2021). Our results offer insights into these complex dynamics, highlighting the importance of understanding the limits of behavioural plasticity and how it interacts with anthropogenic pressures to predict the likely fate of species in our changing environment.

## Author Contributions

**Natasha Gillies:** conceptualization (equal), formal analysis (lead), methodology (lead), writing – original draft (lead), writing – review and editing (lead). **Jack Thorley:** conceptualization (equal), formal analysis (equal), methodology (equal), writing – original draft (supporting), writing – review and editing (supporting). **Henri Weimerskirch:** conceptualization (equal), data curation (equal), funding acquisition (equal), project administration (equal), writing – original draft (supporting), writing – review and editing (supporting). **Stéphanie Jenouvrier:** conceptualization (supporting), data curation (equal), funding acquisition (equal), project administration (equal), writing – original draft (supporting), writing – review and editing (supporting). **Christophe Barbraud:** data curation (equal), funding acquisition (equal), project administration (equal), writing – original draft (supporting), writing – review and editing (supporting). **Karine Delord:** data curation (equal), funding acquisition (equal), project administration (equal), writing – original draft (supporting), writing – review and editing (supporting). **Samantha C. Patrick:** conceptualization (equal), data curation (equal), formal analysis (supporting), funding acquisition (equal), methodology (equal), project administration (equal), supervision (lead), writing – original draft (supporting), writing – review and editing (supporting).

## Conflicts of Interest

The authors declare no conflicts of interest.

## Figures and Tables

**FIGURE A1:**
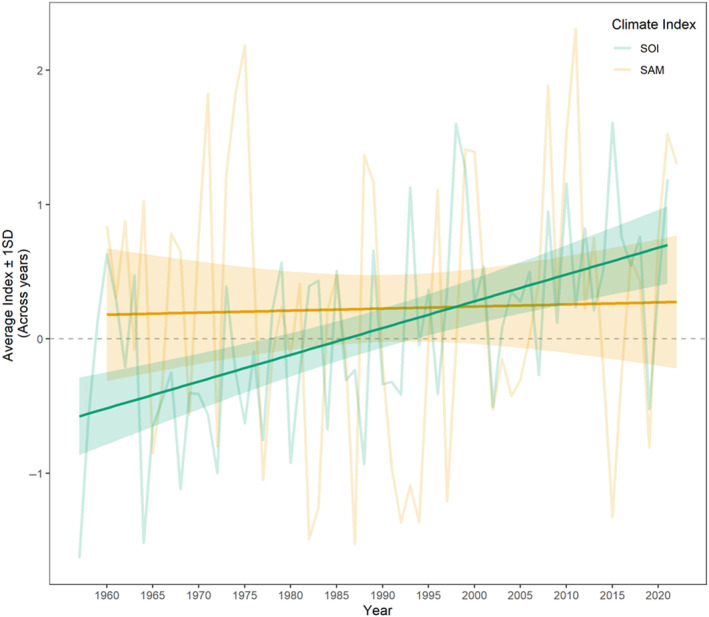
Temporal trends in the Southern Oscillation Index (green lines and shading) and Southern Annular Mode (yellow lines and shading). Lighter lines indicate annual variation in the climate indices, darker lines and shading indicate regression and associated 95% confidence interval of change in index over time.

## Data Availability

All data supporting the results as well as associated scripts to conduct the analyses can be accessed on Zenodo at https://zenodo.org/doi/10.5281/zenodo.10887354.

## Appendix 1

### Climate Variables

The Southern Annular Mode is one of the most descriptive measures of climate variability in the southern hemisphere, explaining 30% of variability in climate (Marshall 2003; Fogt and Marshall 2020). Broadly speaking, SAM influences the north–south movement and strength of a band of westerly winds that encircles the south pole, which has downstream consequences for sea surface temperature, upwelling, and wind speed. Data on SAM indices were accessed from the National Center for Atmospheric Research Climate Data portal (https://legacy.bas.ac.uk/met/gjma/sam.html) on 2022‐01‐20 (Marshall 2003). Monthly SAM indices were calculated as numerical values representing the differences in monthly zonal sea level pressure at 40° S and 60° S. The data are observation‐based, collected from six monitoring stations at each latitude.

The Southern Oscillation Index (SOI) is a measure of large‐scale fluctuations in air pressure between the western and eastern tropical Pacific (between approximately the dateline, and 120° W). SOI typically fluctuates over a year‐to‐year basis but is strongest in December to April. Fluctuation in SOI is associated with periodic sea surface temperature warming in the Pacific Ocean and additionally gives an indication of the intensity of El Niño (warming phase) or La Niña (cooling phase). Monthly SOI indices were downloaded from the National Weather Service—Climate Prediction Center on 2022‐01‐20 (National Weather Service—Climate Prediction Center 2022). SOI is calculated as the standardised difference between anomalies in sea level pressure between Tahiti and Darwin, Australia, normalised by the monthly standard deviation. Positive values generally indicate La Niña conditions, while negative values typically indicate El Niño conditions.

### Assessing Correlations Between SOI and SAM

To assess potential correlations between SAM and SOI, we first fitted ARIMA models to the trend in SAM and SOI over time separately, allowing us to account for temporal structure in the data. We used the R package forecast (Hyndman and Khandakar 2008) to fit ARIMA models. We then extracted the residuals of these models and calculated the Pearson's correlation between them. The correlation between the residuals was very low at −0.012 suggesting that there is not significant annual correlation between SAM and SOI after accounting for their respective temporal structures (Figure A1).

### Foraging Variables

We aimed to calculate metrics of foraging behaviour that would be most likely to be shaped by climate. We focused on four metrics: the number of landings per day of the foraging trip, the ratio of time spent in search relative to travel, the total distance covered over the trip in kilometres, and the median distance covered between searching bouts. These measures were selected based on prior literature that illustrates their capacity to depict how birds respond to their environment, indicate individual‐level variation, and highlight their likely significance as foraging characteristics (Weimerskirch et al. 2007; Patrick, Pinaud, and Weimerskirch 2017; Gillies et al. 2023). Landings are thought to be associated with prey capture attempts and therefore should be a good measure of foraging effort (Weimerskirch, Wilson, and Lys 1997; Weimerskirch and Guionnet 2002). Individual landings were identified as transitions from search to rest states. Ratio of search versus travel time indicates relative investment in exploiting patches of food versus commuting between patches (Patrick, Pinaud, and Weimerskirch 2017; Gillies et al. 2023) and so represents trade‐offs in investment in two competing foraging styles. The ratio was determined by calculating the proportion of each trip spent in each behaviour and dividing these by one another. Total distance covered is highly correlated with prey capture and trip duration, and consequently has been used as a proxy for overall foraging effort in previous studies (Salamolard and Weimerskirch 1993; Weimerskirch et al. 1993). This measure was calculated as the summed distanced between individual GPS points across the entire foraging trip. Finally, distance between foraging patches should indicate how frequently birds were encountering feeding sites and was calculated as the median distance covered (in km) between bouts of search, as identified through by the HMM.

### Effects of Climate on Minimum Latitude

We found evidence that females experienced suboptimal foraging conditions during positive SAM phases, with females exhibiting increased travel behaviour, reduced landings, and increased distance travelled between searching bouts. Positive SAM phases impact more southerly areas to a lesser extent, and so females could theoretically forage further south to abate the impacts of SAM on their foraging. To test whether this was the case, we examined whether the minimum latitude within searching areas was affected by SAM. We fitted a generalised linear mixed model to minimum latitude with the fixed effects of monthly SAM, boldness, and their interaction, and age. We included individual ID and year as random effects. Models were fitted to both males and females, separately.

We found no evidence that minimum foraging latitude was affected by SAM (Table A1), suggesting that despite the poorer conditions in their foraging regions, females were not adjusting their foraging location to compensate.

**TABLE A1:** Model estimates from set of generalised linear mixed effects models examining the effect of the Southern Annular Mode (SAM) and Southern Oscillation Index (SOI) on foraging behaviour.

Response	Predictor	Estimate (odds ratio^a^) and 95% confidence interval		*z* value		*p*	
		*F*	*M*	*F*	*M*	*F*	*M*
Median latitude	SAM	0.00 [−0.00, 0.01]	0.00 [−0.00, 0.01]	0.90	0.64	0.37	0.52
	Boldness	−0.00 [−0.01, 0.01]	−0.00 [−0.01, 0.01]	−0.44	−1.16	0.66	0.25
	**Age**	**−0.00 [−0.00, 0.00]**	**−0.00 [0.00, 0.00]**	**−2.28**	**3.23**	**0.022**	**0.001**
	SOI	1.00 [1.00, 1.01]	1.00 [0.99, 1.00]	1.17	−1.73	0.25	0.084
	Boldness	1.00 [0.99, 1.01]	1.00 [0.99, 1.00]	−0.46	−1.15	0.64	0.25
	**Age**	**1.00 [1.00, 1.00]**	**1.00 [1.00, 1.00]**	**−2.47**	**3.22**	**0.014**	**0.001**

*Note:* Total path distance was fitted with a Gamma distribution; ratio search: Travel with a beta distribution, and landings per day with a Poisson distribution: Estimates are provided on the relevant link scale. An odds ratio of 1 suggests no effect, an odds ratio > 1 suggests a positive association, and an odds ratio < 1 suggests a negative association. Significant *p* values and associated beta estimates and test statistics are highlighted in bold if they were found to be significant in both sexes; they are highlighted in bold and italics if they were found to be significant in only one sex. Non‐significant interactions were dropped.

^a^ Estimate for number of landings is an Incidence Rate Ratio due to Poisson distribution.
